# How to Assess the Efficacy of Interventions for Actinic Keratosis? A Review with a Focus on Long-Term Results

**DOI:** 10.3390/jcm10204736

**Published:** 2021-10-15

**Authors:** Theresa Steeb, Anja Wessely, Anne Petzold, Lutz Schmitz, Thomas Dirschka, Carola Berking, Markus V. Heppt

**Affiliations:** 1 Department of Dermatology, Universitätsklinikum Erlangen, Friedrich-Alexander-University Erlangen-Nürnberg (FAU), 91054 Erlangen, Germany; theresa.steeb@uk-erlangen.de (T.S.); anja.wessely@uk-erlangen.de (A.W.); anne.petzold@uk-erlangen.de (A.P.); carola.berking@uk-erlangen.de (C.B.); 2 Comprehensive Cancer Center Erlangen-European Metropolitan Area of Nuremberg (CCC ER-EMN), 91054 Erlangen, Germany; 3 Department of Dermatology, Venereology and Allergology, Ruhr-University, 44791 Bochum, Germany; lutz.schmitz@dermpath-bonn.de; 4 Institute of Dermatopathology, MVZ Corius DermPathBonn, 53115 Bonn, Germany; 5 Faculty of Health, University Witten-Herdecke, 58455 Witten, Germany; t.dirschka@centroderm.de; 6 CentroDerm Clinic, 42287 Wuppertal, Germany

**Keywords:** actinic keratosis, follow-up, surveillance, long-term efficacy, core outcomes, prevention, squamous cell carcinoma, methodology

## Abstract

Actinic keratoses (AK) are common lesions of the skin caused by cumulative sun exposure. Since AK may progress to invasive cutaneous squamous cell carcinoma (cSCC), guidelines uniformly recommend early and consequent treatment. A variety of interventions are available; however, most randomized controlled trials, meta-analyses, and guidelines focus on outcomes that are usually evaluated 8–12 weeks after the end of treatment. Importantly, these assessments can capture the short-term, transient outcomes, but do not allow any conclusions about long-term results to be drawn and do not reflect the probability of transition towards cSCC. Until now, few studies have assessed the long-term results of interventions for AK. Indeed, finding the most appropriate end-point and adjunct time point for determining the long-term results of interventions for AK remains a challenge. Here, we provide an overview of the different ways of measuring the efficacy of AK treatments, such as using recurrence rates or sustained clearance rates, and discuss methodological aspects. Furthermore, we highlight the importance of evidence from post-marketing surveillance trials for the detection of efficacy values and safety signals. Additionally, we emphasize that a follow-up period of 12 months might not be sufficient to reflect the long-term results and stress the urgent need for a longer follow-up period and regular risk-stratified surveillance.

## 1. Introduction

Actinic keratoses (AK) are commonly occurring precancerous lesions caused by chronic exposure to ultraviolet (UV) radiation [1,2]. They usually manifest as erythematous and keratotic or scaling plaques with a rough, sandpaper-like surface on sun-exposed areas such as the face, ears, arms, and dorsal hands [2,3]. They are among the most common skin lesions, with a prevalence of up to 60% in Caucasians older than 60 years [4]. In the last decade, a clear increase in AK incidence has been observed. The reasons for this rapid development include chronic UV exposure and the demographic change, with a higher proportion of the population being elderly [5]. Thus, AK are estimated to be among the most common reasons for consulting a dermatologist in Caucasian populations in Europe, North America, and Australia [1,4,6]. Visible lesions may be surrounded by tissue that clinically appears unaltered but that has significant UV-induced histological and genetic abnormalities. This theory has generally come to be known as field cancerization, although an exact clinical definition of the term has still not been established [2,7].

AK are a reliable indicator of chronic UV exposure and the presence of multiple lesions represents a valid biomarker for the development of invasive cutaneous squamous cell carcinoma (cSCC) or basal cell carcinoma (BCC) [8]. Specifically, AK are direct precursors of cSCC, with a capacity for lymphogenic and hematogenic spread. The transformation rates without active treatment are low. A systematic review reported a progression rate of 0.075% per lesion per year, rising to 0.53% per lesion in patients previously affected by non-melanoma skin cancer (NMSC) [9]. However, these rates increase rapidly if further risk factors, such as having a light-skinned phenotype, signs of actinic damage, increased age, or chronic immunosuppression, are present [9,10]. As valid and reliable prognostic factors for the development of AK to cSCC are still not available, the consequent and early treatment of AK is mandatory and recommended by various international guidelines [11,12,13]. However, some guidelines also recommend follow-ups in cases featuring the occurrence of single widely scattered AK lesions per field or affected body area, no hyperkeratosis, or no dynamic progression.

## 2. Interventions for AK

A variety of interventions are available for the treatment of AK—for example, surgery; cryosurgery; ablative laser treatment; or topical medication such as 5-fluorouracil cream, 5-fluorouracil 5% with salicylic acid 10% solution, tirbanibulin 1% ointment, imiquimod cream, diclofenac 3% gel, or photodynamic therapy (PDT) using either aminolevulinate (ALA) or its ester methyl-aminolevulinate (MAL) as photosensitizers. According to the mode of application, interventions can be divided into either lesion- or field-directed approaches [14]. Lesion-specific interventions offer a fast and easy approach for treating isolated lesions, whereas field-directed treatments are preferable for treating multiple AK, as they also address the subclinical changes in an actinically damaged field. There already exist several sound randomized controlled trials (RCT) and systematic reviews or meta-analyses that evaluate these interventions and their combinations [15,16,17,18,19,20,21,22,23]. Most of the currently available meta-analyses have identified 5-fluorouracil formulations and PDT to be the most effective for clearing AK, irrespective of their localization, while PDT was found to be preferable for AK located in non-scalp and non-face areas in a recently conducted subgroup meta-analysis [15,16,17,18]. These results may also be applied to other epithelial lesions [24,25].

However, the vast majority of RCT, meta-analyses, and guidelines focus on outcomes that are usually evaluated 8–12 weeks to approximately 6 months after the end of treatment [16,18,19,20,26,27]. Importantly, these assessments can capture the short-term results, such as lesion clearance, but do not allow any conclusions to be drawn about the true long-term outcomes of the interventions of interest; the latter outcomes also include progression to cSCC, which may even take several years. Moreover, treatments for AK are usually approved by regulatory agencies based on short-term outcomes, such as the clearance after 8 weeks or transient side effects, whereas the more important medical issues are in fact the long-term clearance and irreversible side effects.

## 3. Ways of Measuring Long-Term Efficacy: Recurrence vs. “Sustained” Clearance Rates

There is increasing evidence that AK is a chronic condition showing a variable disease course, where even resolution without any active treatment is possible, although this is very rare [9]. Finding the most appropriate endpoint to determine the long-term results of interventions for AK is a challenge. In general, the efficacy outcomes are reported very heterogeneously and inconsistently in RCT regarding their definition and nomenclature. Outcomes can be regarded as either patient-specific —i.e., the patient (inter-individual trials) or treatment field (intra-individual trials) represents the unit of analysis—or as lesion-specific—i.e., the treated AK lesion is the unit of analysis.

Recently, a core outcome set for AK clinical trials was developed. A core outcome set represents a standardized, consented minimum set of outcomes that should be measured and consequently reported in every trial. The AK-specific core outcome set consists of six specific endpoints: the complete clearance of AK, the percentage of AK cleared (lesion-specific), the severity of adverse events (patient-specific), the patient perspective on effectiveness (patient-specific), the patient-reported future treatment preference (patient-specific), and the recurrence rate [28]. The consensus panel recommends assessing the treatment response at 2–4 months and recurrence at 6–12 months, with the AK rate of progression to cSCC reported whenever long-term follow-up is possible [28]. Thus, when the long-term efficacy of a treatment should be reported remains rather vague, and the ideal time point at which to assess these outcomes has yet to be determined. Reporting the recurrence rates at 6–12 months is rather early, and it is debatable whether this time point represents a valid proxy for long-term treatment effects. Recurrence can certainly occur even after initial complete lesion clearance and also later than 12 months after the end of treatment. Surprisingly, according to the core outcome set, when long-term follow-up is possible, the treatment location-specific incidence of and progression to cSCC should be reported, although this is not required in all studies. Notably, the consensus panel specifying the core outcome set also neither regarded the long-term efficacy of a treatment as essential for trial reporting, nor placed this outcome in the innermost circle of an onion model of this core outcome set. Thus, it still remains unclear as to when and how the long-term efficacy of interventions for AK should be assessed. Beyond that, it can hardly be discriminated whether lesions occurring in the follow-up phase are recurrent preexisting lesions or newly developed AK.

### 3.1. Recurrence Rates

The term “long-term efficacy” is certainly multifaceted and comprises several distinct outcomes, such as the recurrence rates and the “sustained” clearance rates. The recurrence rates are only one potential proxy for long-term efficacy. This rate can be calculated either as a participant- or lesion-specific recurrence rate (Table 1). The participant-specific recurrence rate is defined as the number of relapsing patients divided by the number of patients with complete initial clearance. In contrast, the lesion-specific recurrence rate is defined as the number of relapsing lesions divided by the number of lesions with complete initial clearance. However, when performing a pooled analysis of RCT, this rate does not refer to the intention-to-treat (ITT) population in the denominator of the original RCT. A major problem with calculating or pooling recurrence rates is that they can be subject to a large increment even if only a few lesions or patients show a relapse. Typically, not all AK resolve, nor do all patients achieve the complete clearance of their AK. This issue may be misleading, as the denominator for the placebo arms is usually lower than that for the active interventions. Thus, recurrence rates in general as well as pooled recurrence rates should be interpreted cautiously to avoid over-interpretation. To account for this issue, we advise reporting the raw values for each intervention in addition to the relative recurrence rates, which are typically indicated as a percentage.

### 3.2. Sustained Clearance Rates

Investigating the sustained clearance rates represents another way to dissect the long-term efficacy of treatments. This rate can be assessed either as lesion- or participant-specific. The participant-specific clearance rate is defined as the number of patients or entire treatment fields with clearance after a certain time point (e.g., at least 12 months) divided by all randomized patients. In contrast, the lesion-specific clearance rate is defined as the number of individual lesions with clearance after a certain time point divided by all randomized lesions. As this outcome refers to an ITT population, and in contrast to a pooled analysis for recurrence rates, meta-analysis is possible.

Few recently published studies have investigated and reported sustained clearance rates after longer follow-up periods. Two examples are two identically designed pivotal double-blind trials that investigated the new topical intervention tirbanibulin 1% ointment [29]. Initial participant-specific clearance occurred in 44% of patients in trial 1 and 54% of patients in trial 2. After a follow-up completed at 1 year, the estimated sustained complete clearance was 27% among the 174 patients who had received tirbanibulin 1% ointment and had achieved complete clearance, while the estimated percentage of patients with recurrent lesions was 47%. Another recent study assessed four randomized interventions for AK in a head-to-head comparison and reported the outcomes after 3 and 12 months [30]. Overall, the authors found 5-fluorouracil to be most effective after both time points compared to imiquimod, photodynamic therapy, and ingenol mebutate (IMB; after 12 months: 82.4% vs. 71.0% vs. 49.6% vs. 42.9%). Additionally, 5-fluorouracil was also identified to be the most cost-effective in an accompanying analysis [30,31]. Thus, these studies represent important contributions to the investigation of the long-term efficacy of various interventions for AK, and their outcomes and respective assessment points may be considered a role models for future studies.

### 3.3. Lesion Counts

Another way of assessing the efficacy of interventions for AK is counting the number of lesions before and after treatment [32,33,34,35]. This approach has often been criticized as it does not reflect a reliable form of evaluation, as well as because it shows a rather poor interrater reliability. To this end, many studies have been conducted to investigate how the reliability of lesional counts may be increased and have shown, for example, that a higher interrater agreement was achieved with a small number of lesions. Thus, the limitation and/or segmentation of body areas to reduce their number is advisable if lesion counts are assessed [33]. However, one of the major limitations of counting AK is that this method does not illustrate whether new lesions have occurred and whether persisting lesions have cleared after treatment. Furthermore, there is no definitive evidence that a reduction in visible lesions equates to reducing the patient’s risk of developing cSCC. Thus, counting AK may not be the best means to evaluate the long-term efficacy of interventions for AK; neither may it be a suitable approach when field cancerization or multiple lesions are present.

### 3.4. Integrated Scoring System for Assessing AK

Recent studies by Dirschka et al. and Dréno et al. have indicated that the previous staging of AK, their progression to cSCC, and necessary preventive therapies need to be reconsidered [36,37,38]. Thus, new assessment criteria for classifying AK have been proposed, such as the actinic keratosis field assessment scale (AK-FAS) and the actinic keratosis area and severity index (AKASI). AK-FAS includes the assessment of three criteria: the AK area (the total skin area affected by AK lesions), hyperkeratosis, and sun damage [36]. To determine an AKASI score, the head is divided into four regions (scalp, forehead, left/right cheek ear, chin, and nose). Subsequently, the percentage of the area affected by AK is estimated for each area, and the severities of the three clinical signs of AK are assessed: distribution, erythema, and thickness [37]. Both scores integrate multiple factors, such as the number of lesions, size of the affected area, and localization, and can thereby improve the standardized clinical assessment of AK, both as a baseline assessment before treatment initiation and during the course for the evaluation of the treatment response. However, these scores cannot reflect the extent to which lesions clear and new lesions occur. Moreover, they are only applicable to AK located on the scalp or face.

### 3.5. Status Quo of Long-Term Efficacy

Until now, only a few meta-analyses have systematically dissected the long-term results of interventions for AK [39,40,41]. A recent pooled analysis investigated the recurrence rates after at least 12 months as efficacy outcome and found participant-specific recurrence rates to be the lowest, at 39% for cryosurgery and ALA-PDT [40]. In contrast, the highest participant-specific recurrence rates were observed for diclofenac, at 85% [40]. Surprisingly, when examining the lesion-specific recurrence rates, the analysis revealed placebo to have the lowest rate, at 15%, followed by ALA-PDT (20%) and MAL-PDT (34%) [40]. However, these results have to be interpreted cautiously, as AK occasionally undergo spontaneous resolution without any active treatment. Another recent network meta-analysis synthesized the sustained lesion- and participant-specific clearance rates as a proxy for efficacy at least 12 months after the end of treatment [41]. Here, ALA-PDT showed the most favorable risk ratio for the outcomes of participant complete clearance rate and lesion-specific clearance rate [41].

The authors of both long-term evaluations only included RCT in their analyses and chose the time point of at least 12 months after the end of treatment as a proxy for long-term efficacy. The authors justified their choice of this time point as most follow-up studies only report clearance rates after this time and since with increasing time, the chance for loss to follow-up is substantially increasing [41]. Methodically, they referred these rates to the baseline ITT population to account for the initial trial randomization, although the clearance rates were commonly reported in separate follow-up trials. This approach can result in overly conservative estimates, as some patients who have achieved sustained clearance may be lost to follow-up, which then would actually decrease the reported clearance rates.

The other way to examine the long-term efficacy of treatments is to look at the recurrence rates of lesions after complete clearance has occurred. However, reliably determining the recurrence rates requires the continuous and spatially mapped observation of the treated AK to distinguish whether lesions are either relapsing at the same site of origin or whether they arose de novo in adjacent sites. Furthermore, it is debatable whether a 12-month time point represents a valid proxy for long-term efficacy. The time point of 12 months can still be perceived as a measure of short-term clearance, and it is likely too early to capture the risk of progression towards cSCC, which may even take several years. Thus, it remains arguable whether the outcome of a 12-month clearance is a valid proxy or whether it should rather be considered to still be a short-term outcome. Until now, only a few studies that have investigated later time points have been published [42,43,44,45,46]. Nevertheless, investigating later time points inevitably results in a gray area in which clearance rates and secondary prevention overlap and in which treatment-induced clearance is extremely difficult to assess.

This brings up the question of which time points should ultimately be considered when investigating the long-term outcomes of interventions for AK. AK are regarded as precursor lesions for cSCC and thus as a chronic condition requiring lifelong surveillance and treatment. Indeed, the progression of AK towards invasive cSCC is presumably slow, underlining the paramount importance of monitoring outcomes for at least 1 year after active treatment and even beyond. Hence, the most clinically relevant readout for the long-term efficacy of interventions should preferably be the prevention of cSCC formation, rather than simply achieving lesion clearance that lasts for only a few months.

Importantly, systematic reviews and meta-analyses often set the inclusion criteria to RCT due to methodological considerations and to incorporate evidence with the highest possible quality. On top of the gold-standard RCT, however, observational studies with large sample sizes and long observation periods, including phase IV trials, post-marketing surveillance studies, and non-interventional studies, are also valuable sources of data on efficacy and safety outcomes that may provide real-world evidence [47]. The recently published post-marketing surveillance trials LEIDA 1 and 2 compared diclofenac 3% gel to imiquimod 5% cream regarding their long-term clinical outcomes for 36 months [42]. The primary endpoints were the treatment-induced inhibition of histological change to grade III AK and the occurrence of invasive cSCC in the treated areas. Importantly, grade III AK do not represent a marker of severity in the sense that the progression risk is higher; thus, the choice of this endpoint remains questionable. Moreover, these endpoints require a histopathologic assessment of lesions, which may not be performed in other trials, as well as patient care outside of trials, as a diagnosis of AK is usually made on clinical grounds. Interestingly, the short-term clearance and long-term treatment outcomes were discordant in the LEIDA trials, underlining the importance of using a longer follow-up period even after initial clearance [42]. Additionally, a histological assessment of the same lesion is not possible in long-term studies, which limits comparison. Moreover, AK may vary within the biopsied lesion substantially. Overall, inter-rater reliability is not very good in either clinical or histological studies [32]. Therefore, the target parameter of avoiding invasiveness is better and more useful for evaluating long-term efficacy. Nevertheless, observational studies such as post-marketing surveillance trials and non-interventional studies represent an important source of data on the long-term results of interventions for AK, and we advise including evidence from these trials in clinical decision making and guideline development.

## 4. Risk Groups Need Regular Surveillance

Special risk groups such as organ transplant recipients (OTR) and other chronically immunosuppressed people, as well as those undergoing regular dialysis, have an increased risk for the development of AK and cutaneous malignancies. The natural course of AK is less favorable in these high-risk populations, and spontaneous resolution is less likely to occur, as is proposed in immunocompetent populations [19]. Hence, careful post-transplant monitoring with the early and consequent treatment of AK and other precancerous lesions is warranted. Thus, OTR should regularly undergo a professional complete skin examination and be treated if AK become manifest. However, until now no recommendation regarding the appropriate time interval or organization of surveillance exists, so such surveillance needs to be performed according to the physician’s expertise.

Nevertheless, the prevention of new lesions represents an important strategy for this subgroup. Sunscreen and protection against UV radiation are believed to be effective for the chemoprevention of AK. Other approaches include nicotinamide and retinoids such as oral acitretin and isotretinoin [48,49,50]. Moreover, several studies exist investigating the prevention of AK, cSCC, and non-melanoma skin cancer using, for example, conventional or daylight photodynamic therapy in meta-analyses or RCT [51,52]. Both studies demonstrate the efficacy of PDT in the prevention of AK in OTR. However, the sample used in the RCT was relatively small and included only men, which reduces the generalizability of these results.

Other high-risk groups include, for example, patients with two or more keratinocyte carcinomas in the previous 5 years, as investigated in the randomized, double-blinded, placebo-controlled Veterans Affairs Keratinocyte Carcinoma Chemoprevention (VAKCC) trial [46,53]. Here, 932 veterans applied either topical fluorouracil 5% cream (*n* = 468) or vehicle control cream (*n* = 464) to the face and ears twice daily for up to 4 weeks. Besides clearance rates, this trial also investigated as an adjunct outcome the number of participants with ≥1 new AK in 6-month intervals up to 36 months. The calculated incidence rates were consistently higher for the vehicle control cream group than for the 5-fluorouracil group at all time points [53]. Thus, 5-fluorouracil is not just an effective intervention for the initial treatment of AK but also for the prevention of AK up to 36 months.

Surveillance is a significant pillar in the holistic perspective on the treatment of the individual patient affected by AK. Thus, high-risk patients such as OTR should undergo regular surveillance like low-risk cSCC patients.

## 5. Other Important Long-Term Outcomes: Safety and Cosmesis

The long-term safety of the interventions is difficult to assess, as most interventions are accompanied by side effects that are typically transient in nature [54]. They usually occur immediately after or during treatment, for example, patients undergoing conventional PDT oftentimes perceive the treatment as painful. Nevertheless, the side effects rarely persist over several months. In some cases, however, adverse events may lead to permanent restrictions or an impaired appearance after the end of treatment. This especially includes scarring as well as the patient’s cosmetic outcome in general. Cosmesis, defined as the subjective cosmetic appearance of the patient after treatment, is an endpoint that may be evaluated by both observers and patients. Cosmetic appearance is determined by changes in skin texture (e.g., tactile roughness), pigmentation, or scarring. To determine long-term results regarding cosmesis, the occurrence of dyspigmentation (hyper- or hypopigmentation) and an improvement of the global response, both measured as dichotomous outcomes, are possible.

Indeed, adverse events such as the occurrence of skin cancer are of paramount importance and may even lead to withdrawals of established topical interventions, as has been recently shown for IMB gel [55,56]. At the beginning of 2020, the European Medicines Agency (EMA) recommended suspending the use IMB, because a post-marketing analysis revealed a higher occurrence of NMSC with IMB compared to imiquimod 5% cream (3.3% vs. 0.4%) [55,56]. Moreover, a pooled analysis revealed a higher incidence of benign skin tumors in patients treated with IMB in comparison to the vehicle (1.0% vs. 0.1%). Furthermore, a higher incidence of NMSC, including basal cell carcinoma, cSCC in situ (Bowen’s disease), and cSCC was also observed compared with the vehicle in four clinical trials with ingenol disoxate (an ester related to IMB) in 1234 patients (7.7% vs. 2.9% of patients) [55,56].

Although the data have not been published in full until now, the EMA currently recommends suspending marketing authorization for IMB in Europe as a measure of precaution. This underlines the high relevance of post-marketing surveillance trials in the detection of long-term results and safety signals, as well as the use of regular surveillance to monitor such adverse events.

## 6. Conclusions

Identifying the most appropriate endpoint and adjunct time points for determining the long-term results of interventions for AK remains a challenge. Mostly, participant- or lesion-specific recurrence rates or sustained clearance rates after a follow-up of 12 months of treatment are reported in the literature. However, this time frame is insufficient to capture the true long-term efficacy of treatments and the progression rates to cSCC. Thus, prospective trials with longer periods of follow-up are urgently needed to observe the efficacy of AK interventions and monitor irreversible side effects. Certainly, it would be useful to follow up with patients for longer to obtain long-term results. However, this may not be feasible from a practical perspective and also not cost-efficient given the small transformation rate of AK into cSCC in the vast majority of patients. Thus, it is especially important to identify those patients at increased risk for the transformation of AK into cSCC. As reliable prediction tools are currently lacking, future research is urgently required in order to identify better risk-stratified surveillance strategies. Besides this, treatment-resistant AK should be monitored carefully, as they might pose a potentially greater risk of progression to cSCC [57]. Nevertheless, post-marketing surveillance trials represent an important source of evidence in the detection of long-term results and safety signals and should be promoted.

## Figures and Tables

**Table 1:** Definitions of the long-term efficacy outcomes for AK.

Outcome	Unit of Analysis	Definition	Measures of Data Aggregation
Recurrence Rate	Participants Treatment fields	$\frac{\mathrm{number}\;\mathrm{of}\;\mathrm{relapsing}\;\mathrm{patients}}{\mathrm{number}\;\mathrm{of}\;\mathrm{patients}\;\mathrm{with}\;\mathrm{complete}\;\mathrm{initial}\;\mathrm{clearance}}$	Pooled rates
	Single lesions	$\frac{\mathrm{number}\;\mathrm{of}\;\mathrm{relapsing}\;\mathrm{lesions}}{\mathrm{number}\;\mathrm{of}\;\mathrm{lesions}\;\mathrm{with}\;\mathrm{complete}\;\mathrm{initial}\;\mathrm{clearance}}$	Pooled rates
Sustained Clearance Rate	Participants Treatment fields	$\frac{\mathrm{number}\;\mathrm{of}\;\mathrm{patients}\;\mathrm{with}\;\mathrm{clearance}\;\mathrm{after},\;e.g.,\;\mathrm{at}\;\mathrm{least}\;12\;\mathrm{months}}{\mathrm{all}\;\mathrm{randomized}\;\mathrm{patients}}$	Meta-analysis, network meta-analysis
	Single lesions	$\frac{\mathrm{number}\;\mathrm{of}\;\mathrm{lesions}\;\mathrm{with}\;\mathrm{clearance}\;\mathrm{after},\;e.g.,\;\mathrm{at}\;\mathrm{least}\;12\;\mathrm{months}}{\mathrm{all}\;\mathrm{randomized}\;\mathrm{lesions}}$	Meta-analysis, network meta-analysis
AK Count	Single lesions	number of lesions at a certain time before treatment vs. after treatment	Meta-analysis, network meta-analysis
